# Characteristics and In-Hospital Outcomes of Pediatric Traumatic Spinal Injuries in A Referral Trauma Center

**DOI:** 10.30476/BEAT.2021.91333.1275

**Published:** 2021-10

**Authors:** Hamid Rezaee, Ehsan Keykhosravi, Amin Tavallaii

**Affiliations:** 1 *Department of Neurosurgery, Mashhad University of Medical Sciences, Mashhad, Iran*; 2 *Department of Neurosurgery, Akbar children’s Hospital, Mashhad University of Medical Sciences, Mashhad, Iran*

**Keywords:** Pediatric, Spine, Trauma

## Abstract

**Objective:**

To evaluate the characteristics and in-hospital outcomes of traumatic spinal injuries among children admitted to a local trauma center in Iran.

**Methods:**

Patients aged 0-18 years who had been admitted to Shahid Kamyab trauma center for acute traumatic spinal injury (Mashhad, Iran) between 2011 and 2018 were evaluated retrospectively. Various demographic, clinical, radiological, and outcome variables were recorded and analyzed.

**Results:**

A total of 127,300 trauma patients were evaluated and amongst them, 61 children had spinal trauma. The mean age was 11.1 and there was no significant sex preponderance (54% males). Most of the injuries were occurred in summer (34.4%) and the most common trauma mechanism was motor vehicle accidents (55.7%) followed by falling (36.1%). Almost all patients (95.1%) had vertebral fractures, which were in the cervical, thoracic, and lumbosacral area in order to decrease incidence. 67.2% of patients were managed non-surgically. The mean hospital stay was 8.9 days and 82.0% of patients had been discharged with normal motor function.

**Conclusion:**

Pediatric spinal trauma is less studied entity in the field of traumatology due to the lower prevalence of these injuries in pediatric patients worldwide. But our study shows a higher prevalence of such injuries in the pediatric population. Although controversial, the leading cause of these injuries is motor vehicle accidents. Fortunately, short term in-hospital outcome seems to be good in such injuries.

## Introduction

Spinal injuries are less common in children but are clinically different from such injuries among adults [1, 2]. The incidence of spine injuries and fractures in children is about 1-9% of all spine injuries [2]. The incidence of acute cervical spine injuries among children is reported to be 1-3%. Derakhshanrad and colleagues showed that only 6.7% of traumatic spinal cord injuries occurred in children from 2011–2015 in Iran [1]. Hospital stay, injury severity, Glasgow Coma Scale (GCS), and the mortality due to traumatic spinal injuries worsen by age [3-5]. Since pediatric spinal injuries are less prevalent, few physicians have good experiences to diagnose and manage these injuries. There is 19% misdiagnosis rate reported in the management of acute cervical spine injuries and fractures in children [6]. Unfortunately, it seems that despite the low incidence rate of traumatic spinal injuries in children worldwide, there is a considerably higher prevalence in Iran. Yet, there are shortage of studies that investigate a specific prevalence and outcomes of spinal injuries amongst children in Iran [1]. This unfortunate higher prevalence in our country comes with the opportunity of investigating this entity in a higher sample-sized study which can yield more statistically reliable results. Therefore, this study was designed to evaluate the characteristics and in-hospital outcomes of traumatic spinal injuries among Iranian children admitted to a referral trauma center in the northeast of Iran.

## Materials and Methods

A retrospective study of patients aged 0-18 years’ old who suffered traumatic spinal injury admitted to the Shahid Kamyab Hospital, a level-one trauma center in Mashhad, Iran was conducted. Patients who were admitted after acute injuries between March 2011 and March 2018 (eight years) were selected for the study. Details were obtained from medical records. The ethics approval was waived by the ethics committee because of the retrospective nature of our study which is basically conducted on the available data and does not include the active participation of patients, and also does not reveal the identity and individualized data of the patients.

The exclusion criteria were children with minor spinal injuries who had been admitted to the emergency department and discharged without any neurosurgical consultation, patients who had been admitted to the emergency department for observation following injuries, patients who had been admitted to another surgical or medical unit after suffering a non-spinal injury, patients who had been visited in outpatient clinics only, and neonates who had suffered birth-related trauma.

Recorded data included age, sex, date of admission and discharge, mechanism of injury, details of injury based on imaging features, GCS, nature of the injury, other coexisting injuries, the requirement for endotracheal intubation, and neurosurgical management. The mechanism of trauma was registered according to the emergency medical services (EMS) report or history from patient’s parent which ever was accessible. Imaging findings were recorded using radiologist reports if accessible and in case of any report’s lack, a radiologist was consulted to report available imaging investigations considering the patient’s available history. GCS was scaled as the first recorded score upon entrance to the hospital or immediately before intubation if pre-hospital intubation was required. Trauma severity was classified as mild (GCS: 13-15), moderate (GCS: 9-12), and severe (GCS: 3-8). Surgical procedures that were performed by other surgical units such as scalp laceration repair performed by the plastic surgeons or long-bone fixations were not included for analysis.

## Results

A total of 127,300 trauma patients had been evaluated between 2011 and 2018 and 215 of them were under 18 years old. Out of 215 children, 61 (28%) had spinal trauma. The mean age was 11.1±4.6 and the majority of children were between 10 to 14 years of age. Male and female were almost equally inflicted with spinal trauma (male=54%)

Pediatric traumatic spinal injuries constituted 0.05% of all traumatic injuries during the study period in our center, but it accounted for 28% of all pediatric traumatic injuries which is a considerably high rate. The total incidence of traumatic spinal injuries was estimated at 7.1 per million children per year. 

Most of the injuries were occurred in summer (34.4%) and the most common mechanism of trauma was motor vehicle accidents (55.7%) followed by falling (36.1%). Twenty-two patients were involved in a car accident and the most common passenger location was in the rear normal seat (16 out of 22). A majority of patients (83.6%) had a GCS of more than 12 at admission time and the cervical range of motion was more than 45 degrees in 73.8% of patients. Six patients (9.8%) had decreased O^2^ saturation, 13.1% of patients had respiratory distress, and 16.4% had concomitant chest trauma. Sixty-four percent of patients had spinal tenderness in cervical, lumbosacral, and dorsal areas, respectively.

Almost all patients (58 patients, 95.1%) had vertebral fractures, which were in the cervical (41%), thoracic (34%), and lumbosacral (24%) spine. The ligamentous rupture was seen in 11 patients and 17 patients had other accompanying fractures. Table 1 describes the patients’ demographic and basic characteristics at the time of admission.

**Table 1:** The patients’ basic characteristics and trauma-related variables

**Variable**s	**N=61**
Age, years	11.1±4.6
Below 5 years	8 (13.1%)
5–9 years	11 (18%)
10–14 years	25 (41%)
15 years and above	17 (27.9%)
Gender	
Male	33 (54.1%)
Female	28 (45.9%)
Season	
Spring	14 (23.0%)
Summer	21 (34.4%)
Autumn	11 (18.0%)
Winter	15 (24.6%)
Injury mechanism	
Motor vehicle accident	34 (55.7%)
Car accident	22 (36.1%)
Front seat with parents	4 (6.6%)
Front seat	2 (3.3%)
Back seat	16 (26.2%)
Bicycle	1 (1.6%)
Motorcycle	5 (8.2%)
Vehicle-pedestrian	6 (9.8%)
Falling	22 (36.1%)
Sharp objects	4 (6.6%)
Sport-related	1 (1.6%)
Glasgow coma scale	
13–15	51 (83.6%)
9–12	9 (14.8%)
≤8	1 (1.6%)
Accompanying conditions	
Decreased O_2_ saturation	6 (9.8%)
Respiratory distress	8 (13.1%)
Chest trauma	10 (16.4%)
Signs of skull base fracture	4 (6.6%)
Spinal tenderness	
Cervical	18 (29.5%)
Dorsal	5 (8.2%)
Lumbosacral	16 (26.2%)
Unable to measure	17 (27.9%)
No tenderness	5 (8.2%)
Cervical range of motion	
≥45°	45 (73.8%)
<45°	8 (13.1%)
Unable to measure	8 (13.1%)
Traumatic Injuries^a^	
Vertebral fracture	58 (95.1%)
Other fractures	17 (27.9%)
Ligamentous injury	11 (18%)

A majority of patients (67.2%) were managed non-surgically and the most commonly used brace was Philadelphia (19 patients). The mean hospital stay was 8.9±9.7 days and 82% of patients had been discharged with normal neurological status. All patients had been discharged with a GCS of more than twelve. Three patients had experienced early postoperative complications, which were wound infection (one patient), bedsore (one patient), and occipital pressure ulcer due to inappropriate use of brace (one patient). Table 2 describes the patients’ management and outcomes.

**Table 2:** The patients’ management and outcomes related variables. SOMI: Sternal Occipital Mandibular Immobilizer

**Variables**	**N=61**
Intervention	
Non-surgical	41 (67.2%)
Surgical	20 (32.8%)
Brace	
Philadelphia	19 (31.1%)
Jewett	12 (19.7%)
Lumbosacral LSO^a^	8 (13.1%)
Halo	4 (4.4%)
SOMI^b^	2 (3.3%)
Minerva	1 (1.6%)
Hospitalization period, days	8.9±9.7
Less than 5 days	27 (44.3%)
5–10 days	16 (26.2%)
More than 10 days	18 (29.5%)
Post-operative complications	3 (4.9%)
Post-operative motor status	
Normal	50 (82%)
Paraplegic	6 (9.8%)
Hemiplegic	1 (1.6%)
Quadriplegic	1 (1.6%)
Hemiparesis	1 (1.6%)
Quadriparesis	2 (3.3%)

^a^LSO: Lumbo-Sacral Orthosis; ^b^SOMI: Sternal Occipital Mandibular Immobilizer

## Discussion

Our study evaluated 61 pediatric spinal trauma during seven years in a level-one trauma center of Mashhad, Iran. The most prevalent mechanism of trauma was motor vehicle collision (MVC), the majority of patients had a vertebral fracture, and 67.2% of them were managed non-surgically. A majority of patients had a normal motor function at discharge, while 13.1% were plegic and 4.9% had paresis. Traumatic spine injuries are less common among children compared to adults. Derakhshanrad and colleagues performed an epidemiological study during 2011-2015 in Iran and evaluated 1215 traumatic spinal cord injuries. These types of injuries were less prevalent in children compared to other age groups. Only 76 (6.7%) of traumatic spine injuries had age below 15 years [1]. A majority of our patients were also between 10 to 14 years old. Out of 698 patients, 32 (4.6%) had 0-19 years of age with traumatic spinal fractures due to motor vehicle accidents in an epidemiologic study by Wang and colleagues in China [2].

Stawicki *et al*., [4] evaluated a total of 6065 injured children by a motor vehicle accident in national datasets during four years and 176 of them (2.9%) were cervical spine injuries. Cervical spinal injuries were more common among girls (56.8%) compared to the boys (43.2%) and in the 13-15 years’ age group more than other age groups. The vehicle type and model and the seating type were not statistically different between the patients with and without cervical spinal injuries. Similarly, a retrospective study evaluated 897 severely injured pediatric patients in an urban level one trauma center during 18 years and 28 of them (18 males and 10 females) had spinal injuries. Mechanism of injuries was falling (35.7%), motor vehicle (32.1%), pedestrian (14.3%), motorcycle (7.1%), horse (7.1%), and ski (3.6%) accidents, respectively. Upper spinal injuries were more common in younger compared to older children and lumbar injuries were only seen in adolescents. Patients were received non-surgically (54%), surgically (32%), and only palliative (14%) treatments. The most common injuries associated with spinal injury were thoracic (89%) and brain (64%) injuries [7]. Galvin *et al*., [8] also evaluated the etiology of spinal cord disease during 10 years among 103 pediatric patients. The majority (66%) of spinal cord diseases were non-traumatic, while it was due to traumatic spinal injury in thirty-five patients (34%). The mechanism of trauma in traumatic spinal injuries was due to motor vehicle accidents (54%), sports injuries (23%), falls (20%), and assault (3%), respectively. They reported an incidence rate of 3.8 per million children for traumatic spinal cord injury during their study period in Victoria, Australia. The most common mechanism of trauma was fall in Özkan et al.’s study (24 out of 75 patients) followed by motor vehicle accidents (21 out of 75 patients) [9]. As can be seen, there is inhomogeneity in the results of studies related to mechanisms of spinal injury but the majority of them advocated motor vehicle accidents as the leading responsible mechanism followed by falls in this population which is in agreement with the results of this study. 

Nitecki and colleagues evaluated the factors associated with outcomes of spine injuries in 227 children. A majority of their patients had lower cervical spine injuries (73%). Cervical spine injuries were more common in younger children. All mortalities (8.4%) were amongst patients with cervical injuries at level C4 or higher. All patients with atlantoaxial fracture or dislocation (11 out of 227 patients) died soon after the injury [5]. Saunders and colleagues showed a decreasing pattern in the incidence rate of spinal injuries among white children from 1998-2012, while the non-white children had an increasing rate. But in our study, there were no decreasing or increasing patterns by year during the study period. They also showed that younger patients with traumatic spinal injuries were associated with female gender, injured through sport, and having concomitant traumatic brain injury [10]. 

Chan and colleagues evaluated 365 pediatric severe trauma patients and 18 of them had cervical spine injuries. Patients with injury to the cervical spine had considerably longer hospital stays. The collar complications were observed in 10% of patients and having collar complications was associated with older age, brain injury, lower GCS, and a longer hospital stay [3]. In our study, only one case of pressure ulcer related to bracing was present and this conflicting result may be due to differences in physical specifications of braces used or different schedules of using them.

It is evident that most of the previous studies done on pediatric spinal injuries are focused mostly on cervical spine injuries and less attention is paid to traumatic injuries of other regions of the spine in the pediatric population. In a study on 72 children (5-17 years old) with traumatic brain injury, the incidence rate of traumatic spinal injuries was 7.3 per million children per year, almost similar to our estimation, and the dorsal level was more commonly inflicted compared to the other levels. Almost half of the patients had vertebral fractures and the most common mechanism of injury was motor vehicle accidents (52.1%) followed by sports injuries [11]. Kerttula and colleagues evaluated the long-term outcomes of post-traumatic wedge-shaped vertebral fracture in fourteen young patients (8.8-20.8 years). They followed the patients for at least one year and found 57% disc-degeneration which was associated with endplate damage at the trauma level in 43% of patients [12]. 

Our study estimated the incidence and prevalence of traumatic spinal injuries similar to other previous studies. This prevalence had not decreased or increased by year during the study period. Our study had some limitations regarding the patients’ data which was evaluated retrospectively and unable to access the missed or incomplete data. Also, in our study, we did not follow the patients to elucidate the long-term outcomes of traumatic spinal injuries. Therefore, it seems that there is a significant need for a well-designed prospective cohort study specifically designed for pediatric traumatic spinal injury to act as a baseline for future more comprehensive interventional studies in this field. We also suggest that a well-organized systematic review of studies performed on this subject can shed a light on different aspects of this entity that are still a matter of debate.

Pediatric spinal trauma is a less known and less studied entity in the vast field of traumatology which is due to the lower prevalence of these injuries in pediatric patients worldwide. But as it can be seen, there are studies like this that show higher rates of prevalence of such injuries in the pediatric population. Although controversial, the leading cause of these injuries is motor vehicle accidents similar to adults which should be considered by policymakers to provide safety for this vulnerable age group. Although short-term in-hospital outcome seems to be good in such injuries, studies with longer follow-up duration may be of benefit for more reliable outcome measurements. 

## References

[1] Derakhshanrad N, Yekaninejad MS, Vosoughi F, Sadeghi Fazel F, Saberi H (2016). Epidemiological study of traumatic spinal cord injuries: experience from a specialized spine center in Iran. Spinal Cord.

[2] Wang H, Liu X, Zhao Y, Ou L, Zhou Y, Li C (2016). Incidence and pattern of traumatic spinal fractures and associated spinal cord injury resulting from motor vehicle collisions in China over 11 years: An observational study. Medicine (Baltimore).

[3] Chan M, Al-Buali W, Charyk Stewart T, Singh RN, Kornecki A, Seabrook JA (2013). Cervical spine injuries and collar complications in severely injured paediatric trauma patients. Spinal Cord.

[4] Stawicki SP, Holmes JH, Kallan MJ, Nance ML (2009). Fatal child cervical spine injuries in motor vehicle collisions: Analysis using unique linked national datasets. Injury.

[5] Nitecki S, Moir CR (1994). Predictive factors of the outcome of traumatic cervical spine fracture in children. J Pediatr Surg.

[6] Avellino AM, Mann FA, Grady MS, Chapman JR, Ellenbogen RG, Alden TD (2005). The misdiagnosis of acute cervical spine injuries and fractures in infants and children: the 12-year experience of a level I pediatric and adult trauma center. Childs Nerv Syst.

[7] Hofbauer M, Jaindl M, Höchtl LL, Ostermann RC, Kdolsky R, Aldrian S (2012). Spine injuries in polytraumatized pediatric patients: characteristics and experience from a Level I trauma center over two decades. J Trauma Acute Care Surg.

[8] Galvin J, Scheinberg A, New PW (2013). A retrospective case series of pediatric spinal cord injury and disease in Victoria, Australia. Spine (Phila Pa 1976).

[9] Özkan N, Wrede K, Ardeshiri A, Sariaslan Z, Stein KP, Dammann P (2015). Management of traumatic spinal injuries in children and young adults. Childs Nerv Syst.

[10] Saunders LL, Selassie A, Cao Y, Zebracki K, Vogel LC (2015). Epidemiology of Pediatric Traumatic Spinal Cord Injury in a Population-Based Cohort, 1998-2012. Top Spinal Cord Inj Rehabil.

[11] Álvarez-Pérez MJ, López-Llano ML (2015). Lesion medular traumatica en la infancia y adolescencia en Asturias [Traumatic spinal cord injury in children and adolescents in Asturias]. Rev Neurol.

[12] Kerttula LI, Serlo WS, Tervonen OA, Pääkkö EL, Vanharanta HV (2000). Post-traumatic findings of the spine after earlier vertebral fracture in young patients: clinical and MRI study. Spine (Phila Pa 1976).

